# From Light-Powered Motors, to Micro-Grippers, to Crawling Caterpillars, Snails and Beyond—Light-Responsive Oriented Polymers in Action

**DOI:** 10.3390/ma15228214

**Published:** 2022-11-18

**Authors:** Mikołaj Rogóż, Zofia Dziekan, Klaudia Dradrach, Michał Zmyślony, Paweł Nałęcz-Jawecki, Przemysław Grabowski, Bartosz Fabjanowicz, Magdalena Podgórska, Anna Kudzia, Piotr Wasylczyk

**Affiliations:** Photonic Nanostructure Facility, Faculty of Physics, University of Warsaw, Pasteura 5, 02-093 Warsaw, Poland

**Keywords:** light-responsive materials, liquid crystal elastomers, micro-robotics, soft robotics, actuators

## Abstract

“How would you build a robot, the size of a bacteria, powered by light, that would swim towards the light source, escape from it, or could be controlled by means of different light colors, intensities or polarizations?” This was the question that Professor Diederik Wiersma asked PW on a sunny spring day in 2012, when they first met at LENS—the European Laboratory of Nonlinear Spectroscopy—in Sesto Fiorentino, just outside Florence in northern Italy. It was not just a vague question, as Prof. Wiersma, then the LENS director and leader of one of its research groups, already had an idea (and an ERC grant) about how to actually make such micro-robots, using a class of light-responsive oriented polymers, liquid crystal elastomers (LCEs), combined with the most advanced fabrication technique—two-photon 3D laser photolithography. Indeed, over the next few years, the LCE technology, successfully married with the so-called direct laser writing at LENS, resulted in a 60 micrometer long walker developed in Prof. Wiersma’s group (as, surprisingly, walking at that stage proved to be easier than swimming). After completing his post-doc at LENS, PW returned to his home Faculty of Physics at the University of Warsaw, and started experimenting with LCE, both in micrometer and millimeter scales, in his newly established Photonic Nanostructure Facility. This paper is a review of how the ideas of using light-powered soft actuators in micromechanics and micro-robotics have been evolving in Warsaw over the last decade and what the outcomes have been so far.

## 1. Introduction

A one-millimeter spider that can walk, jump, fly (on a gossamer), spin a super-strong web, catch prey and replicate for millions of generations (adapting along the way to the changing environment, if necessary), not only inspires awe, but also makes us realize how disappointing and, in most cases, futile are our attempts to mimic nature in the small scale. There is a glaring gap between the scale of meters or centimeters, where industrial mechanisms, machines and robots have a well-established footing, and the molecular scale, where we have gained enough understanding to make molecules dance at our will [1]. The smallest practical electrical motors available are approximately 1 mm in diameter, and given drivetrains of similar scale, a centimeter-long solar-powered walker might ultimately be possible [2]. With piezo drives, some spectacular results have been demonstrated, including a several-second, self-sustained flight [3] on the scale of a small dragonfly (albeit with very limited control).

The challenges in building small autonomous mechanisms are threefold:

- How to design the mechanics to overcome all the problems with friction, van der Waals forces, fluid viscosity and moving with very low Reynolds numbers [4]? The laws governing mechanics and motion in the micro-world are different from those we are used to: gravity and the electromagnetic forces are governed by the same constants, but their relations change with scale. As a result, new challenges as well as new opportunities arise: crawling on an upside-down glass ceiling is easy for a millimeter-scale snail robot [5], while a micron-size walker may struggle to lift its legs, glued to the surface by van der Waals forces [6,7];
- How to fabricate motors, gears and other elements on the sub-millimeter and smaller scales, reliably and cheaply?
- How to supply the mechanisms with energy, either remotely or from an onboard source?

For around ten years now, our group at the Faculty of Physics at the University of Warsaw have been trying, with varying success, to approach microscale mechanics and micro-robotics with soft, light-responsive materials: liquid crystal elastomers. In what follows, we present our achievements to date and sketch out possible avenues of future work.

### 1.1. Liquid Crystal Elastomers as Light-Powered Actuators

Apart from traditional engines and gears, soft actuators capable of powering simple mechanisms and machines, driven by various stimuli, have been demonstrated in small scales [8]. In dielectric elastomer actuators (DEAs), a compressible membrane is sandwiched between flexible electrodes that interact via electrostatic forces [9], resulting in large strains, although with very high voltages needed [10]. Another approach to electrically driven actuators uses conjugated, conductive polymers [11], and asymmetric swelling of gels in ionic solutions can generate shape changes as well [12]. Magnetically driven actuators have also been developed, mostly based on soft polymers with suspended magnetic particles and spatiotemporally varying magnetic fields to control them [13,14]. Material deformation can also be induced by chemical reactions, e.g., selective modification of chemical bonds in polymers [15]. Pressure-driven soft actuators rely on structures with spatially varying stiffness, deforming upon expansion of gas- or liquid-filled chambers [16]—they can be scaled down [17], but still need substantial tubing to deliver the pressurized medium.

It was probably De Gennes who, around 1975, first came up with the idea of anisotropic crosslinked polymer networks with embedded orientational order of nematogenic monomers [18]. This, in turn, triggered various attempts towards fabricating, understanding and using stimuli-responsive-oriented polymers, in particular liquid crystal elastomers (LCEs). LCEs are solid, elastic polymers with a well-defined alignment of molecules (this alignment orientation is often described by the so-called director), just as in the familiar liquid crystals. If cleverly designed, both at the molecular (chemistry) and mesogenic (orientation) level, LCEs may exhibit rather unique mechanical properties, in particular very large, reversible and fast deformations in response to an external stimulus: heat [19]; light triggering photochemical reactions [20,21]; light inducing photo-thermal heating [19,22,23]; electric field [24,25] or the presence of a solvent [26].

**Figure 1:**
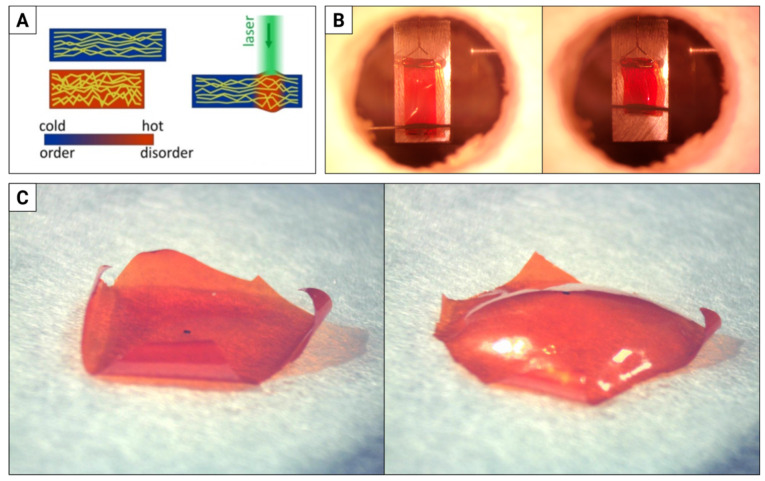
Light-responsive LCE—from a simple contracting strip to films with arbitrary molecular alignment. (**A**) A piece of material with cross-linked polymer chains (olive) arranged in one direction undergoes a phase change in response to temperature increase, either in the entire volume (left) or locally, when illuminated with a green laser beam (right): the molecular order decreases, the polymer chains effectively shorten along the director and macroscopic deformation results. (**B**) A 10 mm long strip of LCE film heated in an oven from 29 °C to 150 °C shortens by approximately 35%. (**C**) A 22 × 22 mm piece of flat LCE film with azimuthal director orientation (the black dot marks the center of rotational symmetry) buckles when heated on a hot plate from 22 °C to 80 °C.

Due to the tight packing of rod-shaped molecules, arranged in cross-linked polymer chains, the order (anisotropic) <-> disorder (isotropic) transition reduces the effective chain length along the alignment direction (the director), at the same time increasing the spacing between the molecules in the perpendicular directions (Figure 1A). As a result, the material deforms in a manner determined by: (a) the spatial distribution of the director; (b) the spatio-temporal distribution of the stimulus, e.g., light intensity; (c) the properties of the material itself (stiffness, elasticity, thermal conductivity) and (d) the surrounding environment (e.g., in our LCEs, the response will usually be different in the air (good thermal insulator) and under water (good thermal conductor)). If stimulated by light, additional degrees of freedom in the mechanical response may involve the light spectrum [27,28] or polarization [29,30]. Thus, LCE elements of various sizes and forms [31,32,33] can be used as micro-actuators in various configurations as well as remotely powered “muscles” for robots (Figure 1B, cf. [34] for a review of LCE actuators and [7] of LCE soft robots).

From the material science and chemistry perspective, LCEs can be categorized by the synthesis pathways—the monomer may be a precursor and a synthesis from low-molar-mass LC is carried out, or a polymer chain (macromer) is grown first and then the material is crosslinked. In terms of the crosslinks’ density, they can be loosely crosslinked liquid crystal polymers, called elastomers, or a highly crosslinked polymer network. As for the location of the mesogenic moieties, they can be built into the polymer backbone in the so-called main-chain LCE, or covalently bonded with the polymer backbone as side groups in the side-chain LCEs.

Following the first experimental demonstration by Finkelmann et al. [35,36], both main-chain and side-chain LCEs were synthesized via different synthesis routes. In the so-called “Finkelmann method”, elastomers with Si-O in the polymer backbone are obtained, capable of high strains, but hydrolyzation reaction is time-consuming and sensitive to impurities and the presence of oxygen. Much later, the “click-chemistry” found its way into the LCE synthesis. These reactions are not sensitive to oxygen and allow for the control of the LCE properties by tuning the structure of the crosslinked liquid crystal polymer [37,38]. The most notable examples are the Michael addition and thiol-ene reactions, leading to LCEs with mechanically programmable alignment, either by shear thinning (in 3D printing) or by surface anchoring [39]. It is also possible to quickly fabricate LCE’s thin films—in a matter of hours—from low-molar-mass LCs, through acrylate homopolymerization. A side-chain LCE is formed with a relatively high number of crosslinks and the alignment in thin films can be induced by rubbing or photopatterning. Additionally, in these materials a sensitizer (e.g., dyes) can be introduced into the matrix before as well as after polymerization and crosslinking.

There are two approaches to actuate LCEs with light. Additional molecules (e.g., dyes) can be embedded into the matrix that absorbs light of a specific wavelength. Most often azobenzene moieties are used, likely due to the extensive studies related to the photoisomerization of azobenzene itself and azobenzene derivatives since the 1960s. An azo-moiety can be built into the polymer backbone (as a monomer or a crosslinker [40,41,42]) and when exposed to light (typically in the UV band), isomerization occurs (trans molecules, supporting the nematic phase, bend and the order parameter in the polymer matrix decreases). After the irradiation stops, the bent molecules go back to their previous state, yet this reversal is slow (it can be sped up with exposure to visible light). Because of the slow response of these reactions (seconds to minutes), some light-responsive LCEs make use of molecules or nano-particles that absorb light and generate heat (which, in turn, reduces the order parameter and induces conformational changes) [43]. Usually, they are dispersed in the LCE matrix: dye molecules, carbon nanotubes [44] or gold nanoparticles [45,46], but coating an LCE actuator surface with NIR-absorbing polymers also works [47]. A combination of photochemical and photothermal responses in light-responsive liquid crystal polymer networks for soft actuators was also presented [48].

### 1.2. LCE Fabrication in Different Scales

LCE elements and structures are often fabricated by UV light-induced polymerization of the liquid monomer, crosslinker and photoinitiator mixture, typically in a glass cell, tens of micrometers thick. Properly prepared orienting surfaces—either rubbed mechanically or coated with photo-orienting layers—guarantee the director orientation within the LCE film in 2 or 2.5 dimensions, if the opposing layers have different properties (Figure 1C). Different elements, typically of the order of a few millimeters, can then be cut with a blade or a laser beam [49]. More complex director patterns—and thus film deformations—are available with patterning of the orienting layers [50,51,52]. Another fabrication method involves shaped molds with the director orientation induced by a magnetic field [53]. In extrusion 3D printing, the molecules are oriented by rheological effects while they flow through a small nozzle [54,55]. The most advanced technology so far—3D laser photolithography (direct laser writing)—uses a focused infrared laser beam that is scanned across a drop of liquid monomer, triggering polymerization via two-photon absorption in a volume (voxel) as small as a fraction of a cubic micron [56].

### 1.3. Problems Yet to Be Solved

While several simple devices have been demonstrated with contracting, bending, twisting or even more complex photo-responsive LCE actuators, typically only their basic response to the light stimulus has been studied. At the same time, even monochromatic light (e.g., in the form of a laser beam(s)) offers a number of degrees of freedom that can be used to control the mechanisms: average power; pulse duration; pulse energy and the light intensity spatial distribution. If the development of mechanisms and machines based on photo-responsive elastomers is to extend beyond single contracting or bending strips of a stimulus-responsive material, a better understanding of the photo-mechanical response of LCEs will be needed, including the interplay of the energy flow and heat dissipation with different light pulse durations and the influence of the light spatial intensity distribution.

Our studies of the millimeter-scale LCE actuators [57] hint that: (a) if used in the transient regime, the actuator response time is independent of laser power; (b) for pulsed laser actuation, long laser pulses result in a smaller actuator response, compared to short laser pulses delivering the same energy; this is true down to a certain pulse duration, determined by the time constants of heat transfer to the environment; (c) when part of the actuator is illuminated with a laser beam of constant total power, but of varying size, the photo-mechanical response is independent of the illuminated area.

Two of the biggest problems we have discovered so far are as follows:

- light absorption in the LCE films decreasing over time, mainly due to the light-absorbing dye bleaching; this can be potentially bypassed by using quantum dots or other, more robust absorbers, well known from fluorescence microscopy, where dye bleaching has been studied and addressed for some time;
- the wear and tear of the actuator after many cycles of operation; this calls for further insights into the mechanical properties of the light-responsive elements, probably at the level of polymer chemistry.

## 2. Micro-Motors—Direct Conversion of Light Energy into Mechanical Work

“Can light drive a motor?” was the opening question asked by Ikeda et al. [58] in 2008. Most energy-harvesting systems convert (solar) light energy either to heat (in solar thermal collectors) or to electricity (in solar cells). Direct conversion of light energy into mechanical energy has been demonstrated, from the molecular to macroscopic scales [59,60,61]. Ultimately, this may enable mechanical devices remotely powered with light (delivered either via free space or through optical fibers), where using electrical cables is not possible.

Yamada et al., in [44], demonstrated a light-driven motor using the contraction of an LCE/polyethylene laminated film irradiated with UV and visible light. The motor presented by Geng et al. [62] used a looped strip of hydroxypropyl cellulose film that deformed under humid air to drive a rotating element. The LCE rod described in [63] can roll with either a light beam or a heated surface as the energy source, but it remains an open question if this deserves to be called a “motor”. In a similar fashion, various LCE tubes and helical ribbons have been demonstrated as drives for centimeter-scale light-powered rolling vehicles [64].

Historically, we first developed micro-robots—the water strider, the caterpillar [50] and the snail [5]—only later to realize that the very same caterpillar, if held in place, can make a rotary motor [65] (if shaped into a ring—“eating its tail”) or a linear inchworm motor (if working in a team of two) [52].

### 2.1. Rotary Motor

**Figure 2:**
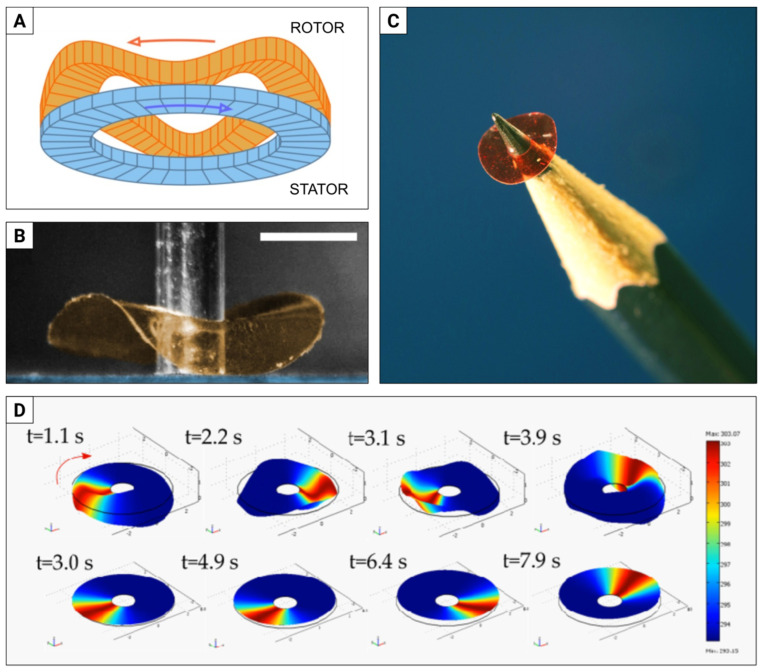
Rotary motor directly powered with light. (**A**) Schematic of the rotary piezoelectric (ultrasonic) motor. Travelling wave deformation generated in the rotor (orange) interacts via friction with the stator (blue) and sets the former in motion. (**B**) LCE rotary micro-motor, seen from the side. The LCE disc (rotor, orange) rotates with respect to the stator (rough solid surface, blue), around a steel axis. The white scale bar is 2 mm long. (**C**) The LCE rotor on a pencil tip for the scale demonstration (although, sadly, we must admit it cannot yet be used to sharpen pencils). (**D**) Numerical simulations of the LCE deformation upon illumination with a spatially modulated (rotating) laser beam. The top row is for azimuthal–radial, and the bottom row for azimuthal–azimuthal director orientation (see text for details). Adapted from [65].

Many high-end camera lenses use so-called ultrasonic motors (USMs) to drive the auto-focus mechanism. Typically, piezoelectric USMs are composed of a ring-shaped rotor and stator (Figure 2A). The voltage applied sequentially to the rotor segments induces travelling wave deformations that couple by friction to the stator, thus setting the former in motion [66,67,68].

Inspired by this design, we built a light-driven micromotor, where the travelling wave deformation results from the photo-mechanical response in a 5.5 mm diameter LCE ring, illuminated with a laser beam moving around the disc circumference (Figure 2B) [65]. In our experiments, we tested LCE discs with different director distributions, in particular, one with the azimuthal orientation on both sides (A-A) and one with the azimuthal and radial orientations (A-R). The A-R disc proved to be 10 times faster (ω = 5.88 rad/min) and 14 times more efficient at converting laser beam revolutions to disc (rotor) revolutions—see [69] for the video of the rotating motor. We also studied these effects by performing finite element numerical simulation, where the dynamic photomechanical response of the LCE discs was modelled as a local strain tensor (Figure 2D).

Despite low speed and efficiency, the LCE micro-rotor has some advantages, such as the ability to be scaled down and to be powered remotely with light energy.

### 2.2. Linear Stepping Inchworm Motor

Even basic understanding of the mechanics of soft materials reveals that it is much easier to build an actuator that generates a pulling force compared to the one that would push using light-responsive LCEs. To extend the portfolio of devices beyond simple LCE strips (often, rather bombastically, pitched as “motors”), we designed and built a linear stepping inchworm motor with two LCE accordion-like actuators [52]. Linear displacement is often a desired mode of operation and various linear motors are in use, either powered from rotary drives or directly from pressure, electromagnetic forces or shape change in different materials. The first inchworm motors had a rotor moved by a sequential action of piezo actuator(s) [70,71]. In a linear inchworm motor, two or more actuators are operated sequentially to push/pull (rather than rotate) a shaft along its axis.

**Figure 3:**
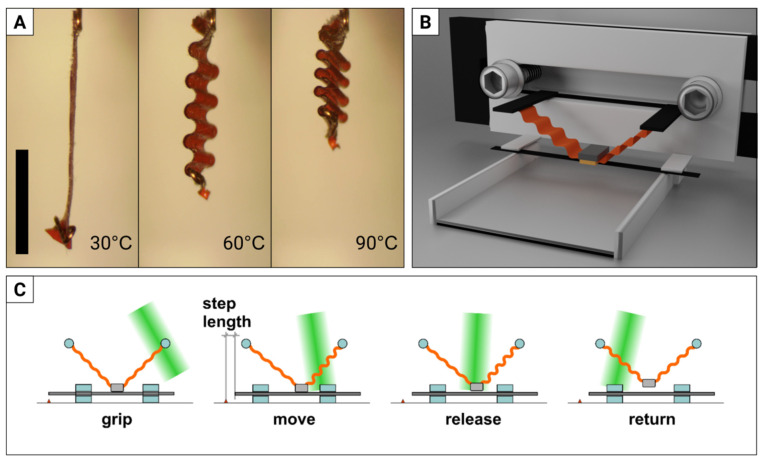
Light-powered linear inchworm motor. (**A**) Accordion-like actuator heated in an oven exhibits very large contraction, with up to 80% stroke. (**B**) CAD rendering of a linear stepping motor with two actuators (orange), gripper (dark grey/yellow) and a sliding shaft (black, under the gripper). (**C**) The sequential action of the actuators, powered by a scanned green laser beam, results in orbital motion of the gripper that, in turn, moves the shaft. The black scale bar in (**A**) is 5 mm long. Adapted from [52].

When fabricating the actuators for our motor, we tested a new LCE-orientation technique that we had conceived a long time before—rubbing overwriting. Mechanical rubbing is commonly used for orienting liquid crystal molecules [72,73]. Our first approach to fabricating LCE films with patterned alignment with rubbing through masks was used to make crawling caterpillars [50], but it required at least two masks and their precise alignment. Here we used a different approach: we rubbed the poly-vinyl alcohol (PVA)-coated glass surface in one direction, then covered certain areas with a mask, and rubbed everything in the perpendicular direction. Accordion-like 50-micron-thick LCE actuators fabricated with this procedure can contract by up to 80% upon heating [50] and were used in the miniature linear motor. A laser beam reflected from a mirror mounted on a galvo scanner illuminates (and thus heats up) two of these actuators, that, in turn, set a small, heavy gripper into an orbital motion (Figure 3B). Each scanning cycle consists of grip–move–release sequence (Figure 3C) and, as a result, the shaft moves (in either direction, defined by the laser scanning direction) at speeds of up to 25 mm/s (see [74] for the video).

The motor design can be straightforwardly adapted to a rotary configuration by replacing the linear shaft with a circular one. If equipped with a position sensor, e.g., optical, the stepping motor can operate in a closed loop configuration, where the length of each step is not relevant, as the distance from the target position is continuously measured and adjusted, including with sub-single-step accuracy.

## 3. Bio-Inspired Millimeter-Scale Robots

For centuries, scientists and engineers have been fascinated by the movement of various animals at different scales and the possibility of copying this movement in man-made machines [75]. Nevertheless, after several decades and huge resources invested in robotics, soft robotics and safe interactions of robots with humans in particular, the results are disappointing: most robots remain awkward automata, at best capable of performing basic repeatable tasks in well-controlled environments (perhaps the latest autonomous cars may soon change this rather gloomy picture). Robotics in the microscale is at an even worse stage—we are nowhere near scaling down robots to millimeters and below, not to mention their reliable fabrication and operation in real-life applications.

### 3.1. The Caterpillar

Conventional robots, made of multiple rigid parts connected by joints, typically have few degrees of freedom and poor adaptability to the environment. One attractive area of research is the realm of soft-bodied animals—segmented worms, mollusks, cephalopods and insects at some development stages (caterpillars)—where using elastic materials could allow their continuous movement to be mimicked, offering the bio-inspired robots the ability to move in confined spaces and to adjust to topologically complex environments [76]. To date, some attempts have been made to replicate them in real scale, with limited success [77].

**Figure 4:**
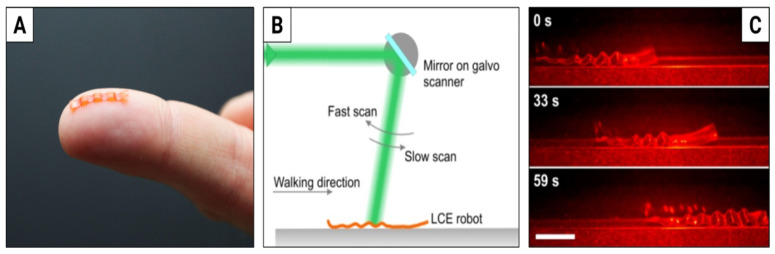
Natural scale crawling caterpillar robot. (**A**) The caterpillar robot on the fingertip of one of its creators. (**B**) Schematic of the experimental setup—the green laser beam is scanned along the robot’s body with a galvo mirror driven by an asymmetric sawtooth signal. The beam was scanned at 0.4 Hz and had 2.5 W of power. (**C**) Snapshots of the video with the light-driven caterpillar crawling on a level surface. The white scale is 5 mm long. The laser light is filtered out with an orange optical filter. Adapted from [50].

Caterpillar locomotion consists of cycles of inching and crawling: the animal lifts and steps forward every pair of its legs—starting from the tail, towards the head. To detach the legs from the ground, it deforms parts of its body, generating a travelling wave of deformation along the body. Such deformations can be induced with spatially varying laser beam in the accordion-like actuators, such as those used in our stepping inchworm motor (compare Figure 3A). Our caterpillar robot [50] was fabricated with a 14.8 × 3.8 mm strip of 50 μm thick LCE film (Figure 4A). When illuminated locally with a scanned green laser beam steered by a galvo scanner (Figure 4B) the film deforms, becomes curved and lifts from the ground, thus generating a wave of deformation. When placed on a rough surface (e.g., sandpaper), this results in crawling locomotion, with a typical step length of 0.3 mm and a maximum speed of 30 mm/min (see Figure 4C for the snapshots and [78] for the video). This is about six times slower than the caterpillar Cucullia verbasci, commonly found in Europe and North Africa [79]. The robot is also able to squeeze through narrow slits, climb a sloped surface and push loads several times its own mass.

With a maximum speed of 30 mm/min, our caterpillar robot may be compared with a few similar demonstrations. In [80], a 29 mm-long robot made of LCE performs inchworm locomotion, powered through cables and reaching 1.91 mm/min. A composite tensegrity robot made of carbon nanotube-doped LCE was able to navigate a labyrinth with the average speed of 6.87 cm/min [81]. Liquid crystal elastomer–carbon nanotube composite was also used in [82] in a crawling and jumping untethered robot, reaching 42 cm/min (half the body length) per minute.

### 3.2. The Snail

**Figure 5:**
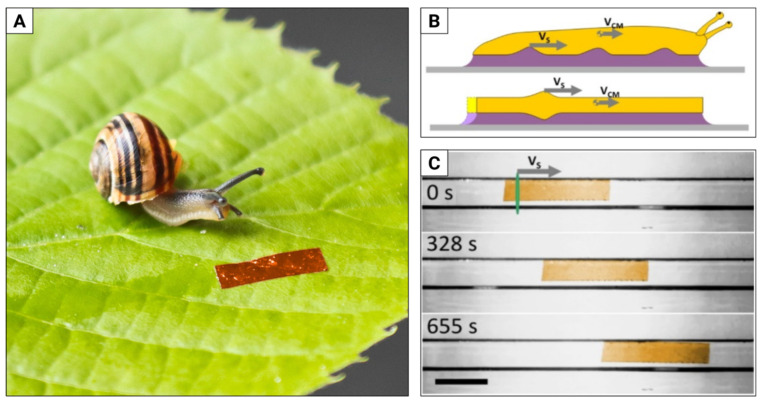
Light-powered snail mimicking the adhesive locomotion of terrestrial gastropods. (**A**) A garden banded-snail Cepea hortensis meets the 10 mm long light-powered snail robot (having no shell, though). (**B**) In snails and slugs, pedal waves propagate along the ventral foot contact surface with a velocity V_S_, propelling the animal with an average speed V_CM_. In a similar way, the light-induced elastomer deformation moves along the robot’s soft body (yellow) covered with an artificial mucus layer (purple). In both cases, the deformations are in fact much smaller. (**C**) Snapshots from a video with the snail robot crawling on a horizontal glass plate topped with glycerine as an artificial mucus. The average speed is 1 mm/min, the black scale bar is 5 mm long. The material contraction was of the order of 0.1 mm. Adapted from [5].

Gastropods—snails and slugs—have a single ventral foot, in which pedal waves propagate, propelling the animal (Figure 5A,B). To further increase the interaction between the foot and the surface, the former is covered with slippery mucus. The (apparently) low complexity of this design and its versatility made snail locomotion a promising target for implementations in robotics.

A 50 μm thick strip of LCE with planar nematic alignment film was placed on a glass substrate covered with a layer of glycerine (which proved to perform best as an artificial mucus) [5]. As with the caterpillar robot, local light-induced deformation was driven by a spatially scanned laser beam, but this time the elastomer contracted along its length, remaining in contact with the glass via the mucus layer. As the laser beam moved, the contraction propagated with a typical speed vs. on the order of several cm/s, resulting in an average robot speed V_CM_ of a few mm/s (see [83] for the video and Figure 5C for snapshots). The robot could move on various surfaces—in the same way as snails—from coarse sandpaper to PTFE (Teflon) [84,85], crawl over a glass tube obstacle, move horizontally upwards (V_CM_ = 0.8 mm/min), downwards (V_CM_ = 1 mm/min) and upside down (V_CM_ = 3 mm/min). Unlike snails that, interestingly, have no reverse gear [86], it could also move backward by reversing the direction of the laser beam scan. In this case, we are not aware of any similar demonstrations of robots performing the mucus-assisted locomotion in natural scale, even though it may be an interesting alternative for robots operating in challenging environments, and it also provides good security margins due to large contact area with the surface.

### 3.3. The Water Strider

Not all our LCE-related projects have resulted in spectacular success. If they had, this would be a clear indication that the challenges were easy to overcome. In fact, a number of the projects have been abandoned, while others were put on hold, even for many years in some cases.

**Figure 6:**
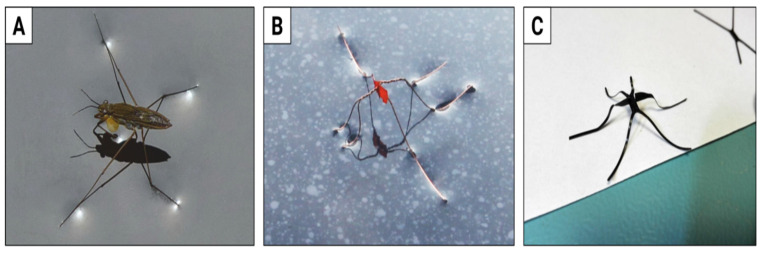
From the pond to the lab (and back)—a light-powered natural-scale water strider robot with LCE muscle. (**A**) Water striders are common inhabitants of reservoirs and rivers in many climate zones. (**B**) First prototype of the light-powered robo-strider with the orange LCE bending actuator powering a pair of legs. (**C**) The latest generation of the robot body (here without the actuator installed) was 2D printed with a 3D extrusion printer and then hot-shaped to take the final form. Water strider photo by Schnobby licensed under CC BY-SA 3.0.

One example of the latter group is the light-powered, natural-scale water strider robot. There has been some substantial effort invested in understanding the dynamics of strider locomotion on the water surface, at some point leading to the so-called Denny paradox (baby striders seemingly move their legs “too slowly” to propel themselves, and yet they still manage to do so) [87], and later to its (apparent) resolution [88]. Striders offer a very good example of animal locomotion to be mimicked in the lab, as they do not need to struggle with friction, capillary or van der Waals forces while moving gracefully on the water’s surface, supported by surface tension (Figure 6A). Our first natural-scale (about 2.5 cm long) robo-strider was built (twisted) from 0.13 mm diameter copper wire, had the simplest muscle-actuator made of a bending strip and had to be guided by a thread, placed just above the water’s surface, to keep on track and remain within the green laser beam powering the LCE actuator (Figure 6B).

Surprisingly enough, it did manage to move, with an average speed of around 180 mm/min—6000 times slower than some members of the Gerridae family. The project has been on hold for a number of years, but has recently re-emerged with a new approach to the strider body—this time with 3D printing (Figure 6C). We hope this will provide a much lighter frame, at the same time offering higher flexibility in the design and repeatability in manufacturing, should we decide to go for a swarm of light-driven water striders one day. As for increasing the speed, there is a class of so-called snap actuators [89], where a piece of material accumulates energy until it reaches a point of nearly instantaneous shape transition, releasing it to the mechanism to be driven (strider legs)—we hope this may be a way towards faster swimming speeds, perhaps one day comparable with wild-born animals.

Other approaches to making robo-striders included a jumping robot with nickel titanium shape memory alloy actuator [90] and a large scale (10 cm long) robot with as many as 12 legs and piezoelectric actuator, reaching the maximum speed of 180 cm/min [91]. As for now, we are not aware of any attempts to involve light-responsive materials in similar constructions.

### 3.4. The Ant

**Figure 7:**
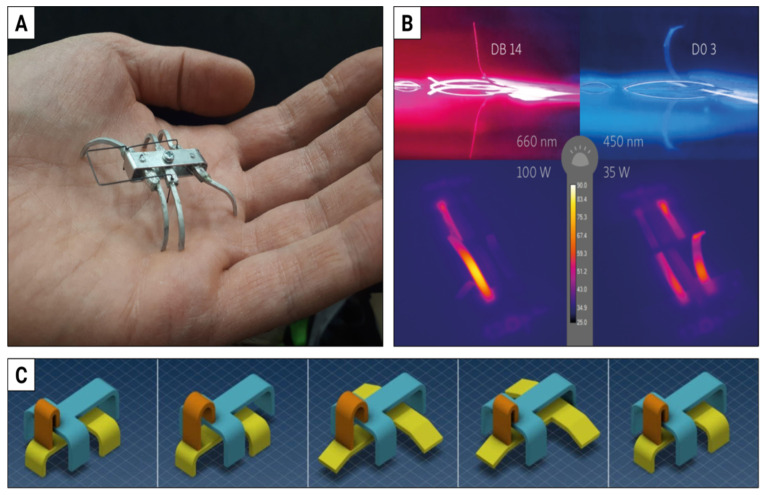
The ultimate walker: LCE ant with two degrees of freedom. (**A**) One of the models built to better understand six-legged locomotion. (**B**) Stripes of LCE with two different dyes, respond by bending to two high-power LEDs with 660 nm and 450 nm centered spectra, respectively. White light (top) and IR (bottom) images. (**C**) One of the concepts of the mechanical design of a six-legged walker with two tripods: one solid (blue) and one with lifting legs (yellow), shifted relative to each other with a bending strip (orange), performing a sequence of deformations that result in a step forward.

The second example of a project awaiting its turn is a millimeter-scale walking ant, powered and controlled by two colors of light, corresponding to the two degrees of freedom in the movement of its six legs. Two separate stimuli, applied in sequence, are the minimum for true non-reciprocal motion [4]. Insects (as opposed to arthropods, for instance) walk on six legs—this seems to be optimized for the number of points of contact with the ground, as they form two tripods that are lifted sequentially and shifted with respect to each other. To better understand this mode of locomotion, we have built several models (see Figure 7A for an example). The next step was to develop two LCE films, responding independently to two different colors of light (Figure 7B). Importantly, the illumination for the millimeter-scale walker must be provided by LEDs, not lasers, as the illuminated area must be large enough to let the walker make at least several steps, before walking into the dark [92]. Ultimately, the mechanical design must take into account many limitations of LCEs, e.g., illumination from one (or two) sides only and the available deformations of the actuators—one concept is presented in Figure 7C.

## 4. Micrometer-Scale Light-Power Tools

Perhaps our failures in mimicking nature, in particular when small scale and/or large quantities of mechanisms are in play, stem from the way we approach fabrication. In the lab, workshop or factory, we would start with materials (a plank of wood, a sheet of metal, a piece of wire, a stretch of foil) and then cut, drill, mill or grind to make individual parts first, and then glue, weld, solder or rivet, to join them together. Obviously, this is not the way things are made in the natural world—there, they grow. This, then, is a question we asked ourselves: can we perhaps grow micro-mechanisms, instead of making them in the traditional way [93]?

**Figure 8:**
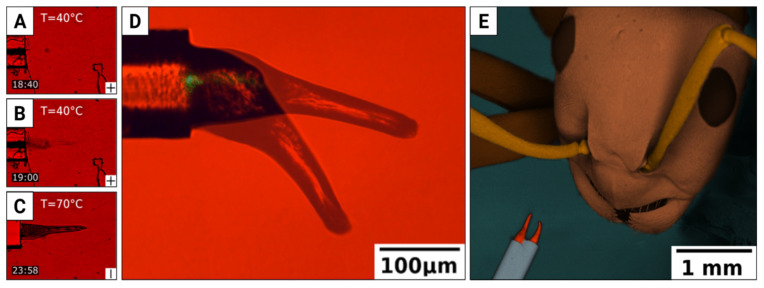
Fiber-grown microscale gripper. (**A**–**C**) Snapshots from the video show the bending actuator growth inside an LC-mixture-filled cell. Time in min: sec is shown in the bottom left corner of each frame. (**D**) Bending actuator in action, powered with green light delivered through the fiber (two photographs have been superimposed to show the magnitude of the deformation). (**E**) Two fibers with bending actuators make a light-powered gripper, here next to a Formica polyctena ant with its mandibles about ten times the size of our tool. Adapted from [93].

To this end, we developed a method of fabricating micrometer-scale elastomer structures with a photo-mechanical response by sending UV light via an optical fiber immersed in an oriented liquid monomer, so that the polymerization occurs at the fiber tip—see Figure 8A–C for the snapshots of the fascinating growth process and [94] for the time-lapse video. In our experiments, a cone-shaped structure grows that bends when visible (green) light is delivered via the same fiber (Figure 8D and [94] for the video).

Gripping objects is fundamental for living organisms and in many machines. Mechanical grippers are typically powered by electric, pneumatic, hydraulic or piezoelectric servos and work well at larger scales, but their complexity and need for transmitting force from a distant servo to the gripper elements prevent their miniaturization and remote control. By joining two fibers with LCE-bending structures, we built a micrometer-scale gripper, powered and operated remotely with light energy delivered through the fibers (Figure 8E).

The gripper, nicknamed “optical pliers” (to avoid confusion with “optical tweezers” [95]), can deliver a gripping force of the order of 10^−7^ N that may be compared, for instance, to a single bending structure weight of approximately 3 × 10^−9^ N (see [94] for the video of the experiment where this force was determined). They do not require any displacement transmission—only energy is transmitted—which makes them very simple and potentially very reliable. In addition, they demonstrate that, with clever technologies, opto-mechanical micro-structures can be fabricated without resorting to any complex (and expensive) microfabrication technology, such as laser photolithography.

Will we one day be able to program and control the growth, so that we can make actuators, or even more complex tools and mechanisms, on fibers, at will? Where would the “genetic information” be stored? In the material, in the growth conditions (molecular orientation, temperature, flow) or in the light that initiates the polymerization—its wavelength(s), polarization, or spatial or temporal modulation? Perhaps in the structure of the optical fiber itself? The concept of “building by growth” involves many more questions than answers and opens up a new, fascinating avenue of research.

## 5. Conclusions and Outlook

Ten years of working and playing with soft, light-responsive materials have taught us many lessons. The most important one is perhaps the lesson of humility, when comparing our results in micromechanics and micro-robotics with the wonders of the natural world that we try to mimic.

At the moment we have several ongoing projects, including the following:

- Orienting LCE with the electric field during laser photolithography [96]. If successful, this technology will open up the ultimate realm of 5D photo-mechanical microstructures: the 3D-printed shape (with sub-micron resolution) with two angles of director orientation, programmable over the entire volume;
- Orienting LCE molecules with writing direction in laser photolithography. This method is somehow similar to orienting by squeezing through a small nozzle in 3D printing of LCEs [97], but on a much smaller scale. As it does not require any photo aligning layers or electrodes, it can be used with minute substrates, such as the end face of an optical fiber;
- Exploring the theme of “materials as machines” [98], we have developed a conveyor belt with LCE-sorting mechanisms, where small objects can be sorted into a number of buckets with LCE stripes (levers) that respond to their different colors and push them from the belt, without any sensors, data processing or separate actuators;
- Since our lab has its origins in photonics and optics, we are constantly playing with ideas of combining LCE structures with optical elements, e.g., optical fibers. We have tested several approaches to fiber switches, either with self-standing light-responsive actuators or with LCE micro-structures grown on the fiber tip.

Liquid crystal elastomers have been pitched as “promising materials” [99] that “open up new horizons in micro actuation and complex, remotely powered and controlled soft-robotics” [50]. Over the last decade, we have learned a lot about their many problems, not least related to repeatable fabrication, reliability and durability. If ever they may find their way into practical applications, the route will be a long one and not without hurdles and pitfalls. However, the journey into the world of light-responsive materials has given us a lot of joy and we hope that some of this is visible in our publications, including this one.

## Data Availability

The datasets used and analyzed during the studies presented are available on reasonable request from the corresponding author.

## References

[1] Kistemaker J.C.M., Lubbe A.S., Feringa B.L. (2021). Exploring Molecular Motors. Mater. Chem. Front..

[2] Challenge for the World’s Smallest|History of Namiki DC Coreless Motor|Adamant Namiki Precision Jewel Co., Ltd. https://www.ad-na.com/en/product/dccorelessmotor/micromotor.html.

[3] Jafferis N.T., Helbling E.F., Karpelson M., Wood R.J. (2019). Untethered Flight of an Insect-Sized Flapping-Wing Microscale Aerial Vehicle. Nature.

[4] Purcell E.M. (1977). Life at Low Reynolds Number. Am. J. Phys..

[5] Rogóż M., Dradrach K., Xuan C., Wasylczyk P. (2019). A Millimeter-Scale Snail Robot Based on a Light-Powered Liquid Crystal Elastomer Continuous Actuator. Macromol. Rapid Commun..

[6] Zeng H., Wasylczyk P., Parmeggiani C., Martella D., Burresi M., Wiersma D.S. (2015). Light-Fueled Microscopic Walkers. Adv. Mater..

[7] Zeng H., Wasylczyk P., Wiersma D.S., Priimagi A. (2018). Light Robots: Bridging the Gap between Microrobotics and Photomechanics in Soft Materials. Adv. Mater..

[8] Hines L., Petersen K., Lum G.Z., Sitti M. (2017). Soft Actuators for Small-Scale Robotics. Adv. Mater..

[9] Rosset S., Niklaus M., Dubois P., Shea H.R. (2008). Mechanical Characterization of a Dielectric Elastomer Microactuator with Ion-Implanted Electrodes. Sens. Actuators A Phys..

[10] Brochu P., Pei Q. (2010). Advances in Dielectric Elastomers for Actuators and Artificial Muscles. Macromol. Rapid Commun..

[11] Bay L., West K., Sommer-Larsen P., Skaarup S., Benslimane M. (2003). A Conducting Polymer Artificial Muscle with 12% Linear Strain. Adv. Mater..

[12] Must I., Kaasik F., Põldsalu I., Mihkels L., Johanson U., Punning A., Aabloo A. (2015). Ionic and Capacitive Artificial Muscle for Biomimetic Soft Robotics: Ionic and Capacitive Artificial Muscle for Biomimetic Soft Robotics. Adv. Eng. Mater..

[13] Lum G.Z., Ye Z., Dong X., Marvi H., Erin O., Hu W., Sitti M. (2016). Shape-Programmable Magnetic Soft Matter. Proc. Natl. Acad. Sci. USA.

[14] Diller E., Zhuang J., Zhan Lum G., Edwards M.R., Sitti M. (2014). Continuously Distributed Magnetization Profile for Millimeter-Scale Elastomeric Undulatory Swimming. Appl. Phys. Lett..

[15] Grinthal A., Aizenberg J. (2013). Adaptive All the Way down: Building Responsive Materials from Hierarchies of Chemomechanical Feedback. Chem. Soc. Rev..

[16] De Volder M., Reynaerts D. (2010). Pneumatic and Hydraulic Microactuators: A Review. J. Micromech. Microeng..

[17] Paek J., Cho I., Kim J. (2015). Microrobotic Tentacles with Spiral Bending Capability Based on Shape-Engineered Elastomeric Microtubes. Sci. Rep..

[18] De Gennes P.-G. (1975). One Type of Nematic Polymers. C. R. Seances Acad. Sci. Ser. B.

[19] Sawa Y., Urayama K., Takigawa T., DeSimone A., Teresi L. (2010). Thermally Driven Giant Bending of Liquid Crystal Elastomer Films with Hybrid Alignment. Macromolecules.

[20] Yu Y., Nakano M., Ikeda T. (2003). Directed Bending of a Polymer Film by Light. Nature.

[21] Braun L., Linder T., Hessberger T., Zentel R. (2016). Influence of a Crosslinker Containing an Azo Group on the Actuation Properties of a Photoactuating LCE System. Polymers.

[22] Pevnyi M., Moreira-Fontana M., Richards G., Zheng X., Palffy-Muhoray P. (2017). Studies of Photo-Thermal Deformations of Liquid Crystal Elastomers under Local Illumination. Mol. Cryst. Liquid Cryst..

[23] Yang H., Buguin A., Taulemesse J.-M., Kaneko K., Méry S., Bergeret A., Keller P. (2009). Micron-Sized Main-Chain Liquid Crystalline Elastomer Actuators with Ultralarge Amplitude Contractions. J. Am. Chem. Soc..

[24] Urayama K., Honda S., Takigawa T. (2005). Electrooptical Effects with Anisotropic Deformation in Nematic Gels. Macromolecules.

[25] Spillmann C.M., Ratna B.R., Naciri J. (2007). Anisotropic Actuation in Electroclinic Liquid Crystal Elastomers. Appl. Phys. Lett..

[26] Urayama K. (2007). Selected Issues in Liquid Crystal Elastomers and Gels. Macromolecules.

[27] Kumar K., Knie C., Bléger D., Peletier M.A., Friedrich H., Hecht S., Broer D.J., Debije M.G., Schenning A.P.H.J. (2016). A Chaotic Self-Oscillating Sunlight-Driven Polymer Actuator. Nat. Commun..

[28] Cheng Z., Wang T., Li X., Zhang Y., Yu H. (2015). NIR–Vis–UV Light-Responsive Actuator Films of Polymer-Dispersed Liquid Crystal/Graphene Oxide Nanocomposites. ACS Appl. Mater. Interfaces.

[29] Tabiryan N., Serak S., Dai X.-M., Bunning T. (2005). Polymer Film with Optically Controlled Form and Actuation. Opt. Express.

[30] Martella D., Nocentini S., Micheletti F., Wiersma D.S., Parmeggiani C. (2019). Polarization-Dependent Deformation in Light Responsive Polymers Doped by Dichroic Dyes. Soft Matter.

[31] Gelebart A.H., Jan Mulder D., Varga M., Konya A., Vantomme G., Meijer E.W., Selinger R.L.B., Broer D.J. (2017). Making Waves in a Photoactive Polymer Film. Nature.

[32] Liu X., Kim S.-K., Wang X. (2016). Thermomechanical Liquid Crystalline Elastomer Capillaries with Biomimetic Peristaltic Crawling Function. J. Mater. Chem. B.

[33] Cheng Z., Ma S., Zhang Y., Huang S., Chen Y., Yu H. (2017). Photomechanical Motion of Liquid-Crystalline Fibers Bending Away from a Light Source. Macromolecules.

[34] Ge F., Zhao Y. (2020). Microstructured Actuation of Liquid Crystal Polymer Networks. Adv. Funct. Mater..

[35] Finkelmann H., Kock H.-J., Rehage G. (1981). Investigations on Liquid Crystalline Polysiloxanes Liquid Crystalline Elastomers—A New Type of Liquid Crystalline Material. Makromol. Chem. Rapid Commun..

[36] Küpfer J., Finkelmann H. (1991). Nematic Liquid Single Crystal Elastomers. Makromol. Chem. Rapid Commun..

[37] Yoon H.-H., Kim D.-Y., Jeong K.-U., Ahn S. (2018). Surface Aligned Main-Chain Liquid Crystalline Elastomers: Tailored Properties by the Choice of Amine Chain Extenders. Macromolecules.

[38] Barnes M., Cetinkaya S., Ajnsztajn A., Verduzco R. (2022). Understanding the Effect of Liquid Crystal Content on the Phase Behavior and Mechanical Properties of Liquid Crystal Elastomers. Soft Matter.

[39] Herbert K.M., Fowler H.E., McCracken J.M., Schlafmann K.R., Koch J.A., White T.J. (2022). Synthesis and Alignment of Liquid Crystalline Elastomers. Nat. Rev. Mater..

[40] Finkelmann H., Nishikawa E., Pereira G.G., Warner M. (2001). A New Opto-Mechanical Effect in Solids. Phys. Rev. Lett..

[41] Sánchez-Ferrer A., Merekalov A., Finkelmann H. (2011). Opto-Mechanical Effect in Photoactive Nematic Side-Chain Liquid-Crystalline Elastomers: Opto-Mechanical Effect in Photoactive. Macromol. Rapid Commun..

[42] Ahn S., Ware T.H., Lee K.M., Tondiglia V.P., White T.J. (2016). Photoinduced Topographical Feature Development in Blueprinted Azobenzene-Functionalized Liquid Crystalline Elastomers. Adv. Funct. Mater..

[43] Mehta K., Peeketi A.R., Liu L., Broer D., Onck P., Annabattula R.K. (2020). Design and Applications of Light Responsive Liquid Crystal Polymer Thin Films. Appl. Phys. Rev..

[44] Marshall J.E., Ji Y., Torras N., Zinoviev K., Terentjev E.M. (2012). Carbon-Nanotube Sensitized Nematic Elastomer Composites for IR-Visible Photo-Actuation. Soft Matter.

[45] Liu X., Wei R., Hoang P.T., Wang X., Liu T., Keller P. (2015). Reversible and Rapid Laser Actuation of Liquid Crystalline Elastomer Micropillars with Inclusion of Gold Nanoparticles. Adv. Funct. Mater..

[46] Hauser A.W., Liu D., Bryson K.C., Hayward R.C., Broer D.J. (2016). Reconfiguring Nanocomposite Liquid Crystal Polymer Films with Visible Light. Macromolecules.

[47] He Q., Wang Z., Wang Y., Wang Z., Li C., Annapooranan R., Zeng J., Chen R., Cai S. (2021). Electrospun Liquid Crystal Elastomer Microfiber Actuator. Sci. Robot..

[48] Lahikainen M., Zeng H., Priimagi A. (2018). Reconfigurable Photoactuator through Synergistic Use of Photochemical and Photothermal Effects. Nat. Commun..

[49] Cheng Y., Lu H., Lee X., Zeng H., Priimagi A. (2020). Soft Actuators: Kirigami-Based Light-Induced Shape-Morphing and Locomotion (Adv. Mater. 7/2020). Adv. Mater..

[50] Rogóż M., Zeng H., Xuan C., Wiersma D.S., Wasylczyk P. (2016). Light-Driven Soft Robot Mimics Caterpillar Locomotion in Natural Scale. Adv. Opt. Mater..

[51] Ware T.H., McConney M.E., Wie J.J., Tondiglia V.P., White T.J. (2015). Voxelated Liquid Crystal Elastomers. Science.

[52] Rogóż M., Haberko J., Wasylczyk P. (2021). Light-Driven Linear Inchworm Motor Based on Liquid Crystal Elastomer Actuators Fabricated with Rubbing Overwriting. Materials.

[53] Yao Y., Waters J.T., Shneidman A.V., Cui J., Wang X., Mandsberg N.K., Li S., Balazs A.C., Aizenberg J. (2018). Multiresponsive Polymeric Microstructures with Encoded Predetermined and Self-Regulated Deformability. Proc. Natl. Acad. Sci. USA.

[54] Wehner M., Truby R.L., Fitzgerald D.J., Mosadegh B., Whitesides G.M., Lewis J.A., Wood R.J. (2016). An Integrated Design and Fabrication Strategy for Entirely Soft, Autonomous Robots. Nature.

[55] Truby R.L., Lewis J.A. (2016). Printing Soft Matter in Three Dimensions. Nature.

[56] Zeng H., Wasylczyk P., Cerretti G., Martella D., Parmeggiani C., Wiersma D.S. (2015). Alignment Engineering in Liquid Crystalline Elastomers: Free-Form Microstructures with Multiple Functionalities. Appl. Phys. Lett..

[57] Grabowski P., Haberko J., Wasylczyk P. (2020). Photo-Mechanical Response Dynamics of Liquid Crystal Elastomer Linear Actuators. Materials.

[58] Yamada M., Kondo M., Mamiya J., Yu Y., Kinoshita M., Barrett C.J., Ikeda T. (2008). Photomobile Polymer Materials: Towards Light-Driven Plastic Motors. Angew. Chem. Int. Ed..

[59] Wang H., Pumera M. (2015). Fabrication of Micro/Nanoscale Motors. Chem. Rev..

[60] Xu L., Mou F., Gong H., Luo M., Guan J. (2017). Light-Driven Micro/Nanomotors: From Fundamentals to Applications. Chem. Soc. Rev..

[61] Chen H., Zhao Q., Du X. (2018). Light-Powered Micro/Nanomotors. Micromachines.

[62] Bisoyi H.K., Li Q. (2016). Light-Driven Liquid Crystalline Materials: From Photo-Induced Phase Transitions and Property Modulations to Applications. Chem. Rev..

[63] Ahn C., Li K., Cai S. (2018). Light or Thermally Powered Autonomous Rolling of an Elastomer Rod. ACS Appl. Mater. Interfaces.

[64] Lu X., Guo S., Tong X., Xia H., Zhao Y. (2017). Tunable Photocontrolled Motions Using Stored Strain Energy in Malleable Azobenzene Liquid Crystalline Polymer Actuators. Adv. Mater..

[65] Dradrach K., Rogóż M., Grabowski P., Xuan C., Węgłowski R., Konieczkowska J., Schab-Balcerzak E., Piecek W., Wasylczyk P. (2020). Traveling Wave Rotary Micromotor Based on a Photomechanical Response in Liquid Crystal Polymer Networks. ACS Appl. Mater. Interfaces.

[66] Morita T. (2003). Miniature Piezoelectric Motors. Sens. Actuators A Phys..

[67] Oh J.-H., Jung H.-E., Lee J., Lim K.-J., Kim H.-H., Ryu B.-H., Park D.-H. (2009). Design and Performances of High Torque Ultrasonic Motor for Application of Automobile. J. Electroceram..

[68] Spanner K., Koc B. (2016). Piezoelectric Motors, an Overview. Actuators.

[69] Traveling Wave Rotary Micromotor. https://www.youtube.com/watch?v=2w5vf_kfO5Y&ab_channel=PNaF.

[70] Smits J.G. (1992). Design Considerations of a Piezoelectric-on-Silicon Microrobot. Sens. Actuators A Phys..

[71] Bexell M., Tiensuu A.-L., Schweitz J.-Å., Söderkvist J., Johansson S. (1994). Characterization of an Inchworm Prototype Motor. Sens. Actuators A Phys..

[72] Montazami R., Spillmann C.M., Naciri J., Ratna B.R. (2012). Enhanced Thermomechanical Properties of a Nematic Liquid Crystal Elastomer Doped with Gold Nanoparticles. Sens. Actuators A Phys..

[73] Han W.C., Sim G.W., Kim Y.B., Kim D.S. (2021). Reversible Curvature Reversal of Monolithic Liquid Crystal Elastomer Film and Its Smart Valve Application. Macromol. Rapid Commun..

[74] Inchworm Motor Based on Liquid Crystal Elastomer Actuators Fabricated with Rubbing Overwriting. https://www.youtube.com/watch?v=PhxzyFMsWJU&ab_channel=PNaF.

[75] Lombardero M., Yllera M. (2019). del M. Leonardo Da Vinci’s Animal Anatomy: Bear and Horse Drawings Revisited. Animals.

[76] Kim S., Laschi C., Trimmer B. (2013). Soft Robotics: A Bioinspired Evolution in Robotics. Trends Biotechnol..

[77] Pilz da Cunha M., Debije M.G., Schenning A.P.H.J. (2020). Bioinspired Light-Driven Soft Robots Based on Liquid Crystal Polymers. Chem. Soc. Rev..

[78] Light-Driven Soft Robot Mimics Caterpillar Locomotion in Natural Scale. https://www.youtube.com/watch?v=mAGK8JG0gVY&ab_channel=PNaF.

[79] Brackenbury J. (1999). Fast Locomotion in Caterpillars. J. Insect Physiol..

[80] Wang C., Sim K., Chen J., Kim H., Rao Z., Li Y., Chen W., Song J., Verduzco R., Yu C. (2018). Soft Ultrathin Electronics Innervated Adaptive Fully Soft Robots. Adv. Mater..

[81] Wang Z., Li K., He Q., Cai S. (2019). A Light-Powered Ultralight Tensegrity Robot with High Deformability and Load Capacity. Adv. Mater..

[82] Ahn C., Liang X., Cai S. (2019). Bioinspired Design of Light-Powered Crawling, Squeezing, and Jumping Untethered Soft Robot. Adv. Mater. Technol..

[83] Snail Soft Robot, Light-Powered, Made of LCE. https://www.youtube.com/watch?v=u3z3TdB4BT0&ab_channel=PNaF.

[84] Denny M. (1980). The Role of Gastropod Pedal Mucus in Locomotion. Nature.

[85] Denny M.W. (1981). A Quantitative Model for the Adhesive Locomotion of the Terrestrial Slug, *Ariolimax columbianus*. J. Exp. Biol..

[86] Parker G.H. (1911). The Mechanism of Locomotion in Gastropods. J. Morphol..

[87] Suter R., Rosenberg O., Loeb S., Wildman H., Long J. (1997). Locomotion on the Water Surface: Propulsive Mechanisms of the Fisher Spider. J. Exp. Biol..

[88] Hu D.L., Chan B., Bush J.W.M. (2003). The Hydrodynamics of Water Strider Locomotion. Nature.

[89] Kim Y., van den Berg J., Crosby A.J. (2021). Autonomous Snapping and Jumping Polymer Gels. Nat. Mater..

[90] Koh J.-S., Yang E., Jung G.-P., Jung S.-P., Son J.H., Lee S.-I., Jablonski P.G., Wood R.J., Kim H.-Y., Cho K.-J. (2015). Jumping on Water: Surface Tension–Dominated Jumping of Water Striders and Robotic Insects. Science.

[91] Song Y.S., Sitti M. (2007). Surface-Tension-Driven Biologically Inspired Water Strider Robots: Theory and Experiments. IEEE Trans. Robot..

[92] Kudzia A. (2021). Liquid Crystal Elastomer Films as Light-Driven Actuators with the Spectral Degree of Freedom. Bachelor’s Thesis.

[93] Zmyślony M., Dradrach K., Haberko J., Nałęcz-Jawecki P., Rogóż M., Wasylczyk P. (2020). Optical Pliers: Micrometer-Scale, Light-Driven Tools Grown on Optical Fibers. Adv. Mater..

[94] Optical Pliers: Micrometer-Scale, Light-Driven Tools Grown on Optical Fibers. https://www.youtube.com/watch?v=j-R-SB6EBqs&ab_channel=PNaF.

[95] Ashkin A., Dziedzic J.M. (1987). Optical Trapping and Manipulation of Viruses and Bacteria. Science.

[96] Carlotti M., Tricinci O., den Hoed F., Palagi S., Mattoli V. (2021). Direct Laser Writing of Liquid Crystal Elastomers Oriented by a Horizontal Electric Field. Open Res. Europe.

[97] Zhang C., Lu X., Fei G., Wang Z., Xia H., Zhao Y. (2019). 4D Printing of a Liquid Crystal Elastomer with a Controllable Orientation Gradient. ACS Appl. Mater. Interfaces.

[98] McCracken J.M., Donovan B.R., White T.J. (2020). Materials as Machines. Adv. Mater..

[99] Wani O.M., Zeng H., Wasylczyk P., Priimagi A. (2018). Programming Photoresponse in Liquid Crystal Polymer Actuators with Laser Projector. Adv. Opt. Mater..

